# Enhanced coherent transition radiation from midinfrared-laser-driven microplasmas

**DOI:** 10.1038/s41598-022-10614-0

**Published:** 2022-05-10

**Authors:** P. B. Glek, A. M. Zheltikov

**Affiliations:** 1 Physics Department, M.V. Lomonosov Moscow State University, Moscow, 119992 Russia; 2 Russian Quantum Center, Skolkovo, Moscow Region 143025 Russia; 3 Department of Physics and Astronomy, Texas A&M University, College Station, TX 77843 USA

**Keywords:** Nonlinear optics, Supercontinuum generation, Terahertz optics, Ultrafast photonics

## Abstract

We present a particle-in-cell (PIC) analysis of terahertz (THz) radiation by ultrafast plasma currents driven by relativistic-intensity laser pulses. We show that, while the *I*_0_ ${\lambda }_{0}^{2}$ product of the laser intensity *I*_0_ and the laser wavelength *λ*_0_ plays the key role in the energy scaling of strong-field laser-plasma THz generation, the THz output energy, *W*_THz_, does not follow the *I*_0_ ${\lambda }_{0}^{2}$ scaling. Its behavior as a function of *I*_0_ and *λ*_0_ is instead much more complex. Our two- and three-dimensional PIC analysis shows that, for moderate, subrelativistic and weakly relativistic fields, *W*_THz_(*I*_0_ ${\lambda }_{0}^{2}$) can be approximated as (*I*_0_*λ*_0_^2^)^*α*^, with a suitable exponent *α*, as a clear signature of vacuum electron acceleration as a predominant physical mechanism whereby the energy of the laser driver is transferred to THz radiation. For strongly relativistic laser fields, on the other hand, *W*_THz_(*I*_0_ ${\lambda }_{0}^{2}$) closely follows the scaling dictated by the relativistic electron laser ponderomotive potential ${\mathscr{F}}_{{\text{e}}}$, converging to *W*_THz_ ∝ ${I}_{0}^{1/2}{\lambda }_{0}$ for very high *I*_0_, thus indicating the decisive role of relativistic ponderomotive charge acceleration as a mechanism behind laser-to-THz energy conversion. Analysis of the electron distribution function shows that the temperature *T*_e_ of hot laser-driven electrons bouncing back and forth between the plasma boundaries displays the same behavior as a function of *I*_0_ and *λ*_0_, altering its scaling from (*I*_0_*λ*_0_^2^)^*α*^ to that of ${\mathscr{F}}_{{\text{e}}}$, converging to *W*_THz_ ∝ ${I}_{0}^{1/2}{\lambda }_{0}$ for very high *I*_0_. These findings provide a clear physical picture of THz generation in relativistic and subrelativistic laser plasmas, suggesting the THz yield *W*_THz_ resolved as a function of *I*_0_ and *λ*_0_ as a meaningful measurable that can serve as a probe for the temperature *T*_e_ of hot electrons in a vast class of laser–plasma interactions. Specifically, the *α* exponent of the best (*I*_0_*λ*_0_^2^)^α^ fit of the THz yield suggests a meaningful probe that can help identify the dominant physical mechanisms whereby the energy of the laser field is converted to the energy of plasma electrons.

## Introduction

Terahertz (THz) radiation is a powerful resource for biomedical applications^1–3^, molecular spectroscopy^3–5^, remote sensing^1,6^, wireless communications^7^, and time-resolved studies of ultrafast charge-carrier dynamics in metals and semiconductors^8–13^. Ultrashort THz field waveforms help coherently control over the dynamics of spins^14^, molecular rotations^9,15,16^, vibrations of crystal lattices^17^, and evolution of free- and bound-state electron wave packets^9–13,18^. In strong-field ultrafast optics, THz generation provides means for particle acceleration^19^, laser-wakefield characterization^20,21^, external-field-assisted high-harmonic generation^11–13,22^, and electron bunch diagnostics at particle-accelerator, synchrotron, and free-electron laser (FEL) facilities^23,24^.

Strong-field optics not only lends a rapidly growing area for applications of THz photonics, but also provides powerful means for high-brightness THz generation, opening ways toward unprecedented levels of THz output energy^25–32^. Standing out as one of the most promising strong-field scenarios for high-yield THz generation is coherent transient radiation (CTR)—secondary radiation emitted by relativistic electrons traversing dielectric discontinuities^33,34^. Laser-driven CTR has been shown to provide a powerful source of THz radiation^25–32,35,36^, enabling the generation of terawatt-level ultrashort pulses of coherent THz radiation with field strengths at the level of 10 GV/m^28,29^ and pulse energies as high as tens of millijoules^30,32^.

Identifying the physical limits of this method of THz generation would provide a deeper understanding of the fundamental aspects of relativistic laser–plasma electrodynamics and would help answer an urgent call of optical science for the development of high-power THz sources. Specifically, the generic *I*_0_ ${\lambda }_{0}^{2}$ scaling of the kinetic energy that electrons tend to pick up from the driver field with intensity *I*_0_ and wavelength *λ*_0_ gives grounds to expect that longer-*λ*_0_ driver pulses, such as those available as an idler-wave output of high-peak power optical parametric chirped-pulse amplifiers (OPCPAs)^37–39^, could help enhance THz generation in relativistic laser–plasma settings. However, relativistic-intensity laser fields can give rise to a complex interplay of concurrent highly nonlinear motions of charged particles in a plasma target. This complex dynamics imprints itself onto a highly sophisticated picture of secondary radiation^27–31,40–44^, giving rise to long-wavelength radiation with widely different spatial, spectral, temporal and polarization properties. The intensity of THz emission in this complex laser–plasma interaction scenario depends on a variety of factors whose relevance to THz generation is sometimes difficult to assess and whose significance is often difficult to quantify. The actual behavior of the THz yield as a function of *I*_0_ and *λ*_0_ in such settings can significantly differ from the naïve *I*_0_ ${\lambda }_{0}^{2}$ expectation^45–48^. Many of the pressing questions related to the brightness scaling of relativistic laser-plasma THz sources, including the scaling of the THz yield as a function of *I*_0_ and *λ*_0_, remain to be answered.

Here, we seek to address these questions by resorting to a particle-in-cell (PIC) analysis of laser–plasma interaction dynamics driven by relativistic-intensity ultrashort laser pulses in a metal-film target. We show that, while the *I*_0_ ${\lambda }_{0}^{2}$ product of the laser intensity *I*_0_ and the laser wavelength *λ*_0_ plays the key role in the energy scaling of strong-field laser-plasma THz generation, the THz output energy, *W*_THz_, does not follow the *I*_0_ ${\lambda }_{0}^{2}$ scaling. Its behavior as a function of *I*_0_ and *λ*_0_ is instead much more complex. Our two- and three-dimensional PIC analysis shows that, for moderate, subrelativistic and weakly relativistic fields, *W*_THz_(*I*_0_ ${\lambda }_{0}^{2}$) can be approximated as (*I*_0_*λ*_0_^2^)^*α*^, with a suitable exponent *α*, as a clear signature of vacuum electron acceleration as a predominant physical mechanism whereby the energy of the laser driver is transferred to THz radiation. For strongly relativistic laser fields, on the other hand, *W*_THz_(*I*_0_ ${\lambda }_{0}^{2}$) closely follows the scaling dictated by the relativistic electron laser ponderomotive potential ${\mathscr{F}}_{{\text{e}}}$, converging to *W*_THz_ ∝ ${I}_{0}^{1/2}{\lambda }_{0}$ for very high *I*_0_, thus indicating the decisive role of relativistic ponderomotive charge acceleration as a mechanism behind laser-to-THz energy conversion. Analysis of the electron distribution function shows that the temperature *T*_e_ of hot laser-driven electrons bouncing back and forth between the plasma boundaries displays the same behavior as a function of *I*_0_ and *λ*_0_, altering its scaling from (*I*_0_*λ*_0_^2^)^*α*^ to that of ${\mathscr{F}}_{{\text{e}}}$, converging to *W*_THz_ ∝ ${I}_{0}^{1/2}{\lambda }_{0}$ for very high *I*_0_. These findings provide a clear physical picture of THz generation in relativistic and subrelativistic laser plasmas, suggesting the THz yield *W*_THz_ resolved as a function of *I*_0_ and *λ*_0_ as a meaningful measurable that can serve as a probe for the temperature *T*_e_ of hot electrons in a vast class of laser–plasma interactions. Specifically, the *α* exponent of the best (*I*_0_*λ*_0_^2^)^α^ fit of the THz yield suggests a meaningful probe that can help identify the dominant physical mechanisms whereby the energy of the laser field is converted to the energy of plasma electrons.

## The physical model and numerical setting

**Figure 1:**
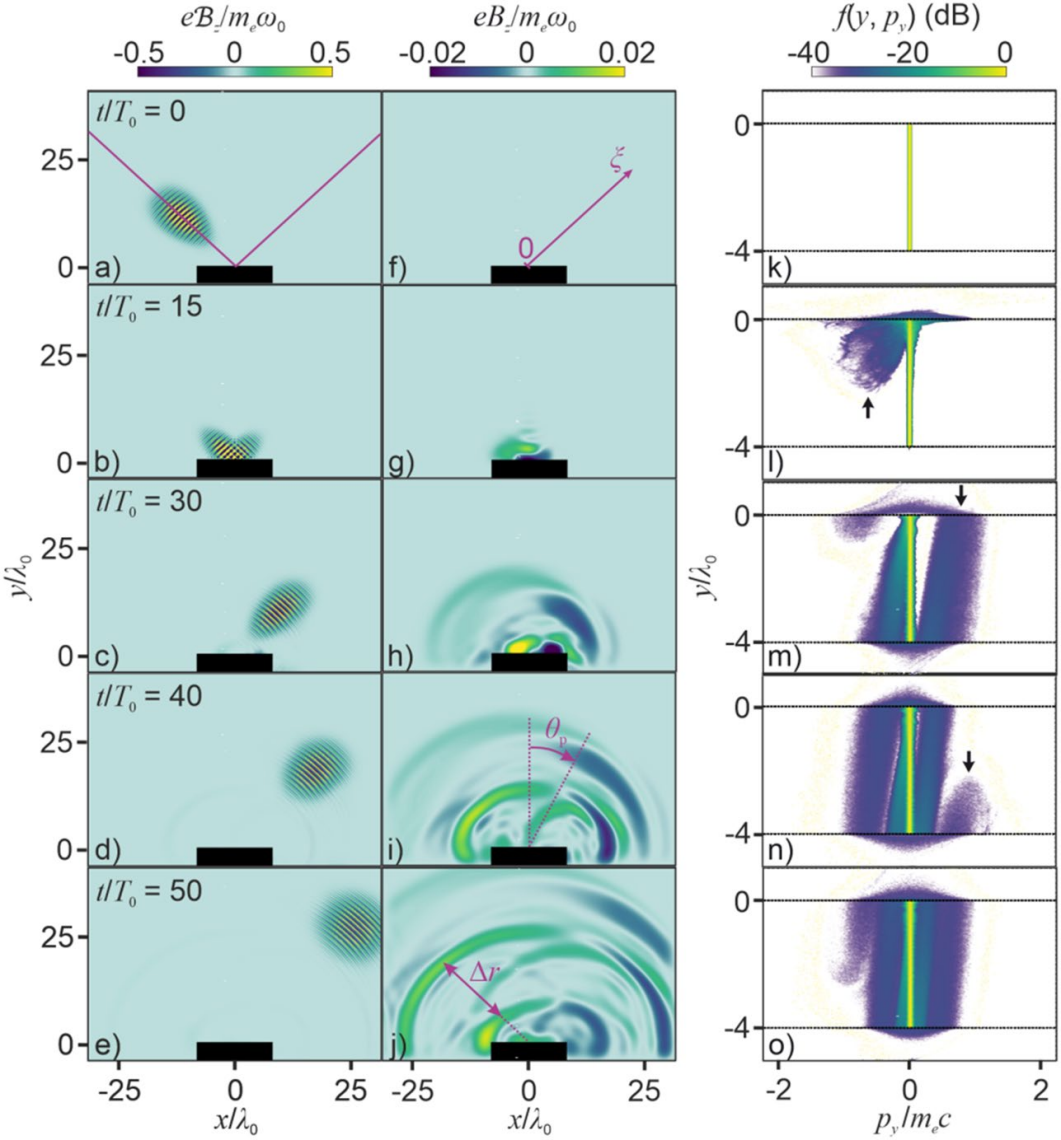
(**a**–**j**) Spatial distributions of (**a**–**e**) the overall field ${\mathscr{B}}$_*z*_(*x*, *y*) and (**f**–**j**) the THz-filtered field ${B}$_*z*_(*x*, *y*) and (**k**–**o**) electron *y*–*p*_*y*_ phase-space distributions *f*(*y*, *p*_*y*_) for a plasma slab with *l*_*y*_ = 4*λ*_0_ driven by a *p*-polarized field with *a*_0_ = 1 and *τ*_0_ = 80 fs at *t* = 0 (**a**, **f**, **k**), 15*T*_0_ (**b**, **g**, **l**), 30*T*_0_ (**c**, **h**, **m**), 40*T*_0_ (**d**, **i**, **n**), and 50*T*_0_ (**e**, **j**, **o**).

We consider a high-intensity laser pulse with a central wavelength *λ*_0_ that drives a plasma slab (Fig. 1a, b) with a width *l*_*x*_ = 16*λ*_0_, a variable depth *l*_*y*_, and initial electron density *n*_0_ = 4*n*_c_, where *n*_c_ = *m*_e_*ω*_0_^2^/(4*πe*^2^) is the critical plasma density, *ω*_0_ = 2*πc*/*λ*_0_ is the central frequency of the laser driver, *c* is the speed of light in vacuum, *m*_e_ and *e* are the electron mass and charge. The initial electron velocity distribution inside the plasma slab is Maxwellian with an electron temperature of 230 eV. Such parameters are representative of plasmas generated on thin solid targets by high-intensity ultrashort laser pulses^40,49–51^.

We simulate the solutions to the Maxwell–Vlasov equations for this laser–plasma interaction setting^40,52^ using a 2D Smilei PIC code^53^. The solutions to the Maxwell equations are computed in this code with the finite-difference time-domain method in a two-dimensional configuration space and three-dimensional momentum space (2D3V) with the electromagnetic field discretized on a Yee grid with space steps Δ*x* = Δ*y* = *λ*_0_/128 along the *x* and *y* coordinates chosen, respectively, within the plane of the target and along the normal to this plane. Each grid cell contains 49 electrons and 49 protons, totaling to over 10^7^ particles of each sort within the entire simulation domain. Time derivatives in the Maxwell equations were computed on a time grid with a step Δ*t* = *T*_0_/256, *T*_0_ being the driver field cycle.

The driver field in our simulations is taken in the form of an ultrashort Gaussian pulse with a pulse width *τ*_0_, an electric field amplitude *E*_0_, central frequency *ω*_0_, and variable polarization. The laser beam makes an angle *φ*_0_ = 45^0^ with the normal to the plasma surface (Fig. 1a, b) and is focused into a beam waist with a diameter *d*_0_ = 4*λ*_0_ on the plasma surface. Providing a meaningful quantifier of the significance of relativistic effects in this laser–plasma interaction setting is the normalized vector potential *a*_0_ = *eE*_0_/(*m*_e_*ω*_0_*c*), which can be viewed as a ratio of the velocity *v*_0_ = *eE*_0_/(*m*_e_*ω*_0_) of nonrelativistic laser-driven electron quiver motion to the speed of light. The driver field is injected into the simulation domain via the Silver–Müller-type boundary conditions. PIC simulations were performed using graphic-processor-unit laboratory clusters^54^, as well as a shared research facility of high-performance supercomputing resources at M.V. Lomonosov Moscow State University^55^.

## THz supercontinua and high-harmonic radiation

As it drives the plasma target, the laser field gives rise to intense radial waves of secondary emission (Fig. 1a–j). The low-frequency part of this radiation field (*E*_*x*_, *E*_*y*_, *B*_*z*_) is isolated in our calculations by applying a super-Gaussian filter ${\mathscr{F}}_{{\text{e}}}$(*ω*) = exp[− (*ω*/*ω*_c_)^*q*^] to the total, driver + secondary radiation field (${\mathscr{E}}$_*x*_, ${\mathscr{E}}$_*y*_, ${\mathscr{B}}$_*z*_) as shown in Fig. 1a–e. We set *q* = 12 and *ω*_c_ = *ω*_0_/4 in calculations presented here to provide a steep passband edge at *ν*_c_ = *ω*_c_/(2*π*) ≈ 20 THz. Fully capturing the field structure of low-*ω* secondary radiation in 2D3V simulations is the *B*_*z*_ field component, whose *xy*-plane maps are shown in Fig. 1f–j.

**Figure 2:**
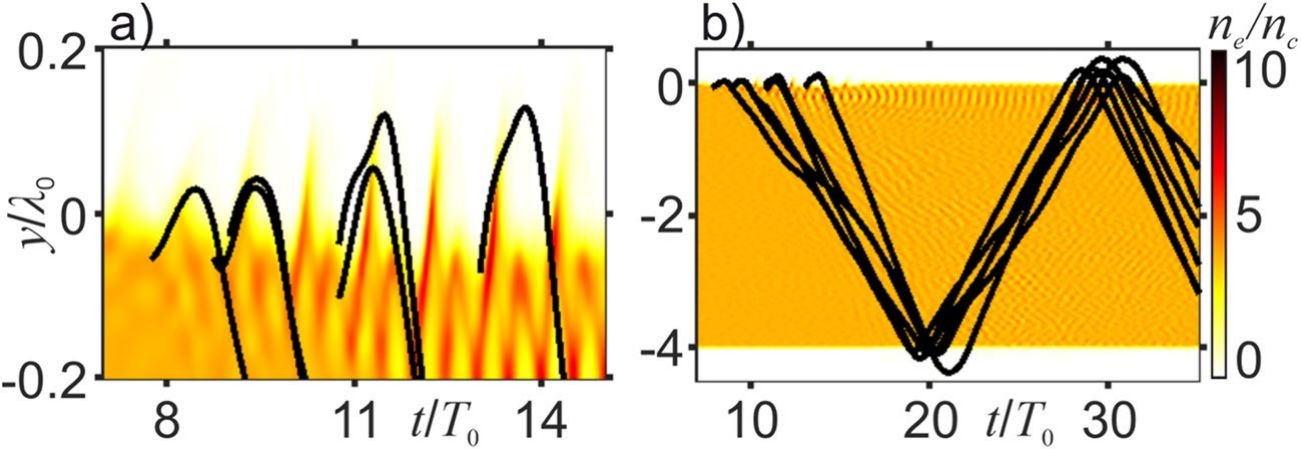
Oscillatory electron *y*(*t*) traces that start at different (*y*, *t*) space–time points, (–0.05*λ*_0_, 7.8*T*_0_), (–0.06*λ*_0_, 8.9*T*_0_), (–0.02*λ*_0_, 8.9*T*_0_), (–0.09*λ*_0_, 10.7*T*_0_), (–0.03*λ*_0_, 10.8*T*_0_), and (–0.07*λ*_0_, 13.0*T*_0_), at the center of the plasma target, *x* = 0, against the map of the electron density, *n*_e_(*x* = 0, *y*, *t*) for *a*_0_ = 1 and *l*_*y*_ = 4*λ*_0_.

As the high-intensity laser pulse reaches the plasma surface at around *t* ≈ 15*T*_0_ (Fig. 1b), the laser field starts to drive plasma electrons, modulating the electron density *n*_e_(*x*, *y*, *t*) inside the plasma and giving rise to signature ripples on the front surface of the plasma target (Fig. 2a). The ponderomotive force induced by the laser pulse drives electron oscillations, making plasma electrons bounce back and forth between the plasma boundaries. In Fig. 2a, b, we follow several oscillatory electron *y*(*t*) traces that start at different (*y*, *t*) space–time points at the center of the plasma target, *x* = 0. Shown against the map of the electron density, *n*_e_(*x* = 0, *y*, *t*), these traces illustrate how the amplitude of electron oscillations tends to grow as the electrons pick more energy toward the trailing edge of the pulse, following a similar behavior of the amplitude of the ripples in *n*_e_(*x* = 0, *y*, *t*).

Each time the laser-driven electrons leave or re-enter the plasma target as a part of their oscillatory motion (e.g., at around *t*/*T*_0_ ≈ 21 and 30 in Fig. 2b), they emit ultrashort bursts of secondary radiation (Fig. 1h–j), giving rise to bright THz supercontinua along with an intense short-wavelength output, dominated by high-order harmonics (HH) of the driver. To calculate the spectrum of this radiation, we choose to work in polar coordinates *r* and *θ*, such that *x* = *r*cos*θ* and *y* = *r*sin*θ*, fix *r* at *r* = 30*λ*_0_, and integrate over the *r* = 30*λ*_0_, *y* > 0 semi-circumference, *S*(*ω*) = ${\int }_{0}^{\pi }|F$[*Q*(*t*, *r* = 30*λ*_0_, *θ*)]|^2^*dθ*, where *F*[∙] is the Fourier transform and *Q*(*t*, *r*, *θ*) = ${\mathscr{B}}$_*z*_(*t*, *r*, *θ*) and ${\mathscr{E}}$_*z*_(*t*, *r*, *θ*) for *p*- and *s*-polarized field, respectively. The spectrum *S*(*ω*) spans the entire THz, infrared, and visible regions, extending deep into the ultraviolet range. In the spatial maps of the THz-filtered field *B*_*z*_(*x*, *y*) (Fig. 1f–j), this process shows up as the first wave of THz radiation, whose emission starts as soon as the driver pulse strikes the plasma surface (cf. Fig. 1b, c, g, h).

**Figure 3:**
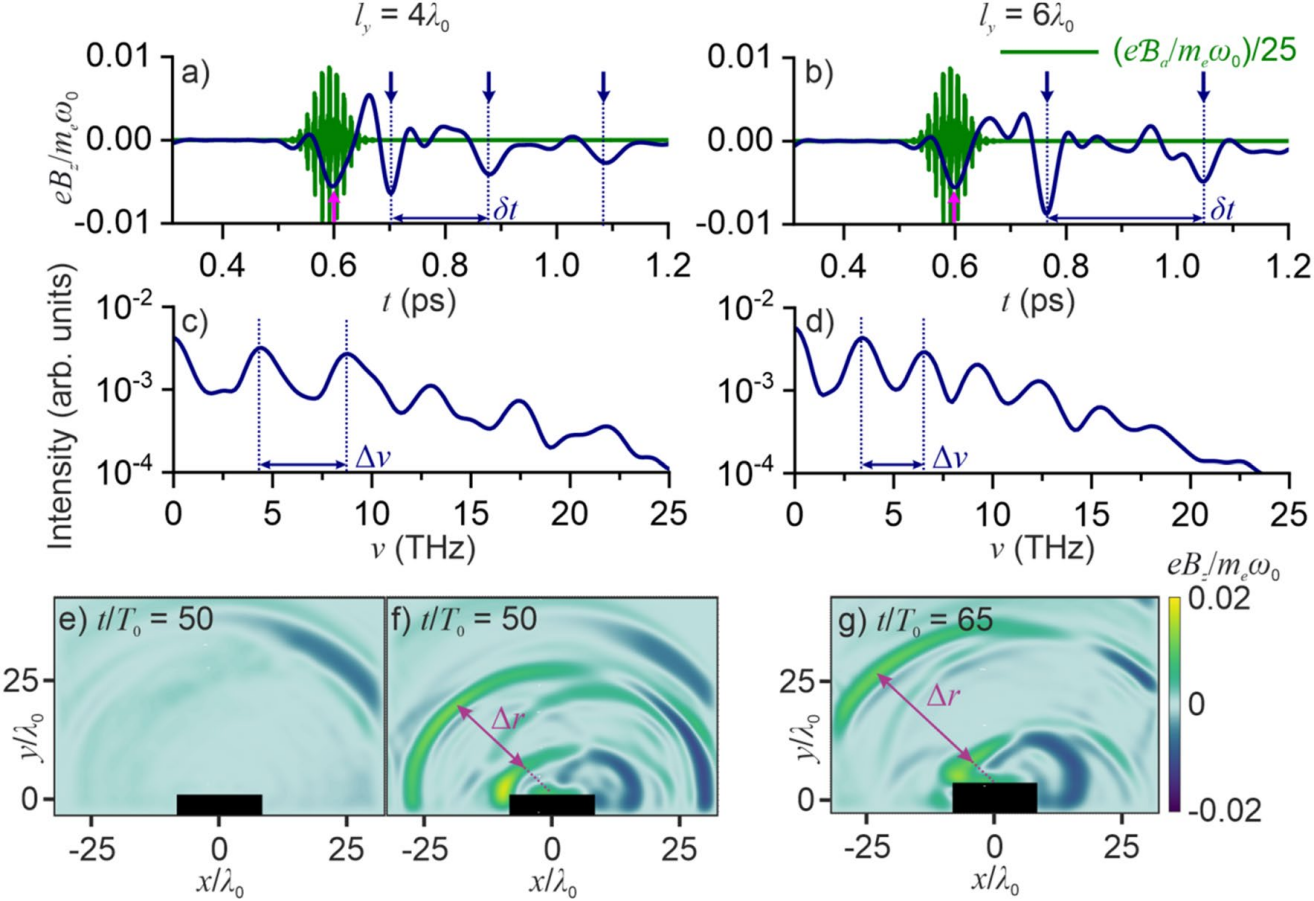
(**a**, **b**) Temporal traces of specular-reflected THz radiation (blue line) and the overall, THz + HH field (green line), (**c**, **d**) spectra of specular-reflected THz radiation, and (**e**–**g**) the snapshots of the spatial distribution of the THz-filtered field *B*_*z*_(*x*, *y*) taken at *t* = 50*T*_0_ (**e**, **f**) and 65*T*_0_ (**g**) for a plasma slab with *l*_*y*_ = 4*λ*_0_ (**a**, **c**, **e**, **f**) and 6*λ*_0_ (**b**, **d**, **g**) driven by a *p*-polarized laser pulse with *a*_0_ = 1 and *τ*_0_ = 80 fs in (**a**–**e**, **g**) a full PIC simulation and (**f**) PIC simulation where electron recirculation is suppressed by halting all the electrons reaching the rear plasma boundary. Shown with arrows in the temporal traces are the instantaneous THz + HH bursts of secondary radiation (pink arrows) and recurring CTR waves (blue arrows).

Presented in Fig. 3a, b are the temporal traces of specular-reflected THz radiation (blue line) and the overall, THz + HH field (green line) whose *xy*-plane snapshots are shown in Fig. 3e, g. The troughs of the first-wave bursts of THz radiation (blue line) are highlighted in these traces with pink arrows. As can be seen from these traces and the spatial field structure, emission of the first THz burst is accompanied by HH generation (HHG). The HH field is emitted in the form of a train of attosecond pulses, tightly confined to the central, most intense part of the laser pulse. Polarization properties of the HH field are in full obedience to the selection rules for relativistic HHG^40,56^—a *p*-polarized driver generates *p*-polarized even- and odd-order HHs; an *s*-polarized driver gives rise to *s*-polarized odd HHs and *p*-polarized even harmonics, and a circularly polarized driver generates even and odd harmonics in both *p* and *s* polarization states.

## Subcycle CTR waves

The spectral properties and the spatiotemporal structure of broadband radiation in Figs. 1, 2 and 3 are understood in terms of a physical scenario that combines CTR by electrons traversing the plasma boundary^23–28,30,31^ with HHG by laser-driven relativistic electrons near the front plasma surface^32,37–39^. In this study, we focus on the THz part of broadband radiation emitted as a part of this complex laser–plasma interaction scenario. Central to THz generation is the ponderomotive force induced by the laser driver, which accelerates plasma electrons, making them move toward the rear surface of the plasma slab. While the laser-driven oscillatory motion of relativistic electrons near the plasma surface gives rise to attosecond pulses of HH radiation, the ponderomotive force induced by the laser driver accelerates plasma electrons, making them move toward the rear surface of the plasma slab. This process is readily discernible in the electron *y − p*_*y*_ phase-space distribution *f*(*y*, *p*_*y*_) as shown in Fig. 1k–o for *a*_0_ = 1. Specifically, at *t* ≈ 15*T*_0_ (Fig. 1l), some of the electrons near the front surface of the plasma slab at *y* = 0 are seen to start gaining momentum from the laser driver (marked with an arrow in Fig. 1l), which has just reached the *y* = 0 plasma surface. Because the laser ponderomotive force pushes electrons toward the inside of the plasma slab, i.e., toward negative *y*, the momentum gained by the electrons in Fig. 1l is negative. Accordingly, at later *t*, the electrons that have picked up their momentum from the laser driver are found deeper within the plasma target (Fig. 1m), with their energy further increased via the action of the laser ponderomotive force.

Near the rear surface of the plasma slab, however, the electrostatic sheath field gradually decelerates and eventually reflects some of these electrons, preventing them from leaving the plasma slab. This effect is readily visible in Fig. 1m, where electrons with positive *p*_*y*_ appear near the rear surface of the plasma slab at *y* = −4*λ*_0_ and move toward its front side, in accordance with the positive sign of *p*_*y*_, building up their momentum and gaining their kinetic energy in the process of this motion (the arrow in Fig. 1m). By the time these electrons reach the front surface of the plasma target at *y* = 0, the energy of some of these electrons is high enough to overcome the binding forces that tend to keep the electrons within the plasma. When such electrons cross the plasma boundary, they emit an outgoing radial CTR wave, clearly visible in Fig. 1i, j. The electrons whose energy is not sufficient to make it to the other side of the plasma boundary are reflected, once again, to continue their circulation within the plasma (the arrow in Fig. 1n). Such recirculating electrons give rise to recurring subcycle bursts of THz radiation in the *y* > 0 semi-space (highlighted with blue arrows in Fig. 3a, b) each time they approach the *y* = 0 plasma boundary with a sufficiently high kinetic energy.

The spatiotemporal structure of the THz field (Figs. 1f–j, 3a, b, e, g) is fully consistent with this picture of THz generation. While the first burst of THz radiation is generated as soon as the driver pulse strikes the plasma surface (cf. Figure 1b, g), the highest intensity of THz radiation in the direction of specular reflection is achieved not within, but in the wake of the laser pulse, at the trough of the first radial subcycle CTR wave (Fig. 1i, j), emitted at *t*_1_ ≈ 0.70 ps (blue arrow in Fig. 3a), as a completion of the first cycle of electron recirculation within the plasma target driven by a laser pulse with *λ*_0_ = 3.9 μm. This first burst of CTR is followed by a second CTR wave, which is observed as a well-resolved subcycle waveform in the temporal trace of the THz field in Fig. 3a with a trough at *t*_2_ ≈ 0.88 ps. As can be seen in the spatial maps of *B*_*z*_(*x*, *y*) in Figs. 1j and 3e, this trough is separated by Δ*r* ≈ 17.5*λ*_0_ from the trough of the first CTR wave. When the thickness of the plasma slab is increased by a factor of 1.5, both the spatial separation Δ*r* between the troughs of the first and second CTR waves (Figs. 3e, 3g) and the delay time *δt* = *t*_2_ − *t*_1_ between these troughs in the temporal trace of the THz field (Fig. 3a, b) increase by a factor of ≈1.5.

**Figure 4:**
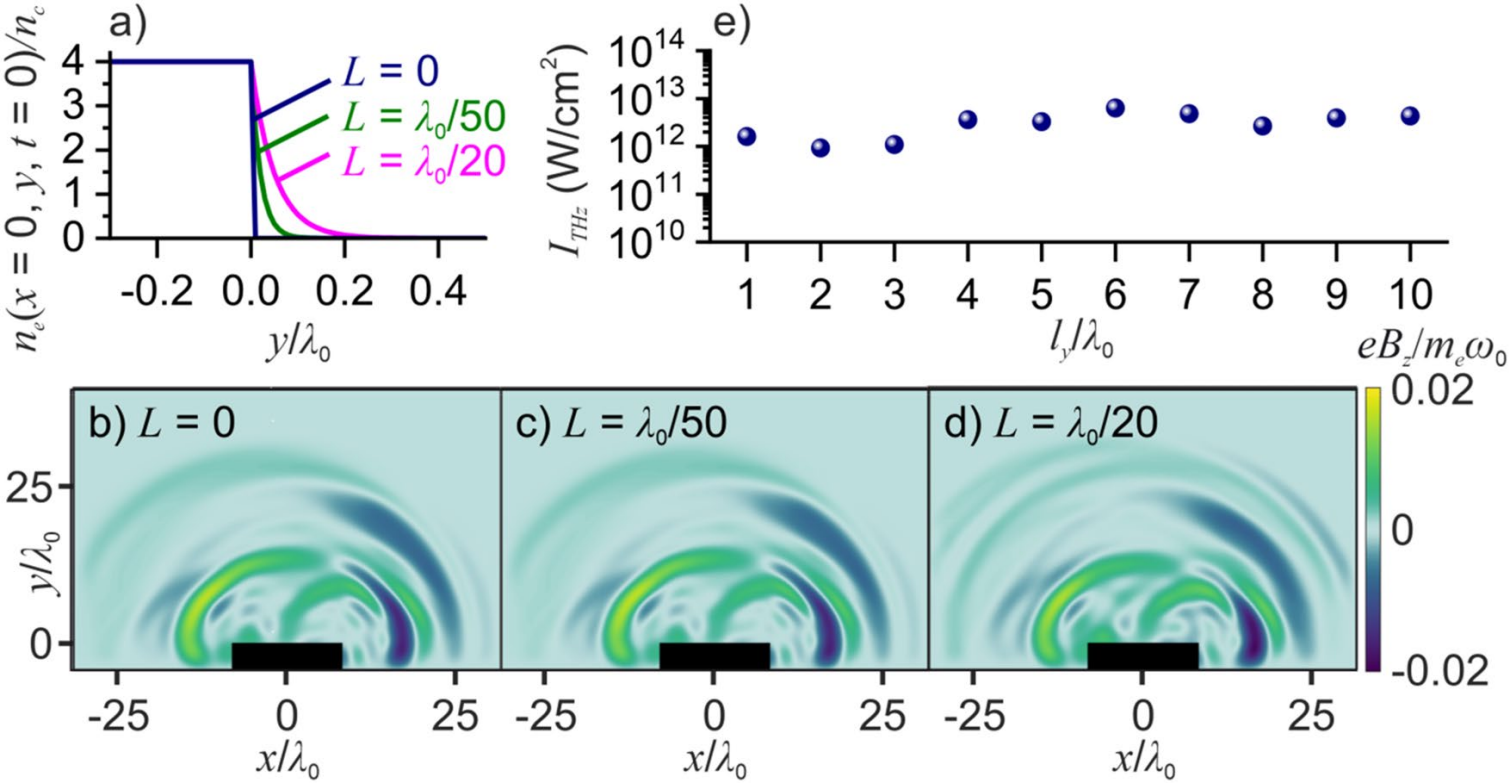
(**a**) Electron density profiles used in simulations. (**b**–**d**) The spatial distribution of the THz-filtered field *B*_*z*_(*x*, *y*) at *t* = 40*T*_0_ for (**g**, **h**) and 65*T*_0_ (**i**) for a plasma slab with *L* = 0 (**b**), *λ*_0_/50 (**c**), and *λ*_0_/20 (**d**) driven by a *p*-polarized laser pulse with *τ*_0_ = 80 fs, *λ*_0_ = 3.9 μm, and *a*_0_ = 1. (**e**) THz output intensity as a function of the plasma depth *l*_*y*_ for a *p*-polarized laser driver with *τ*_0_ = 80 fs, *λ*_0_ = 3.9 μm, and *a*_0_ = 1.

Because THz CTR waves are emitted within time intervals when laser-driven electrons traverse plasma boundaries, the visibility of the spatial crest-and-trough structure of the THz output is sensitive to the sharpness of the plasma boundaries. To quantify this effect, Fig. 4a–d present the spatial maps of the THz radiation field calculated for a plasma slab with the initial electron density profile defined as *n*_*e*_(*x*, *y*, *t* = 0) = *n*_0_ for − 4*λ*_0_ ≤ *y* ≤ 0 and *n*_*e*_(*x*, *y*, *t* = 0) = *n*_0_ exp(− *y*/*L*) for *y* ≥ 0 (Fig. 4a). As the extension of the plasma transition layer *L* (plasma gradient length scale) increases from *L* = 0 to *L* = *λ*_0_/20, the essential features in the crest-and-trough structure of the THz output show little to no variation. This suggests a rather comfortable margin of *L* within which simulations performed within the approximation of infinitely sharp plasma boundaries continue to provide an adequate physical picture of THz radiation. While for gas targets, steep plasma profiles with *L* ≤ *λ*_0_/20 are hard to achieve, in laser–plasma experiments with solid targets, plasma density profiles with *L* ≤ *λ*_0_/100 have been repeatedly demonstrated^57–59^ and even plasma gradients as steep as *L* ≈ *λ*_0_/200 have been achieved^60^.

In the frequency domain, the recurring bursts of THz radiation manifest themselves as clearly resolved fringes in the spectrum of THz radiation (Fig. 3c, d). Unlike the field intensity of the THz laser-plasma output, which is found, in close agreement with earlier studies^31^, to be a very weak function of the plasma depth *l*_*y*_ (Fig. 4e), the spacing between the fringes in THz spectra is defined by the time interval *δt* between the CTR bursts, or the separation Δ*r* between the CTR waves, Δ*ν* = *c*/Δ*r*, and is therefore highly sensitive to *l*_*y*_. Specifically, for a plasma target with *l*_*y*_ = 4*λ*_0_, the CTR waves are separated by Δ*r* ≈ 17.5*λ*_0_, giving rise to fringes with Δ*ν* ≈ 4.4 THz in the spectrum of THz radiation (Fig. 3c). As *l*_*y*_ is increased up to 6*λ*_0_, the spacing between the fringes in the THz spectrum decreases by a factor of ≈1.5, becoming Δ*ν* ≈ 3.0 THz (Fig. 3d).

As a useful verification of the role of recirculating electron currents for THz generation, we performed a series of PIC simulations in which electron recirculation was suppressed by artificially halting all the electrons reaching the rear plasma boundary. Any artificial THz emission that such a halting of electrons may cause does not disturb the THz field outside the plasma, because the plasma, whose plasma frequency is in the 100-THz range in this regime, totally screens such artificial radiation. Specifically, with all the electrons reaching *y* = −4*λ*_0_ brought to a halt, the most intense component of THz radiation, as is readily seen from a comparison of Fig. 3e, f, is completely suppressed.

## The ***I***_0_***λ***_0_^2^ scalability of the THz output and the electron energy distribution function

**Figure 5:**
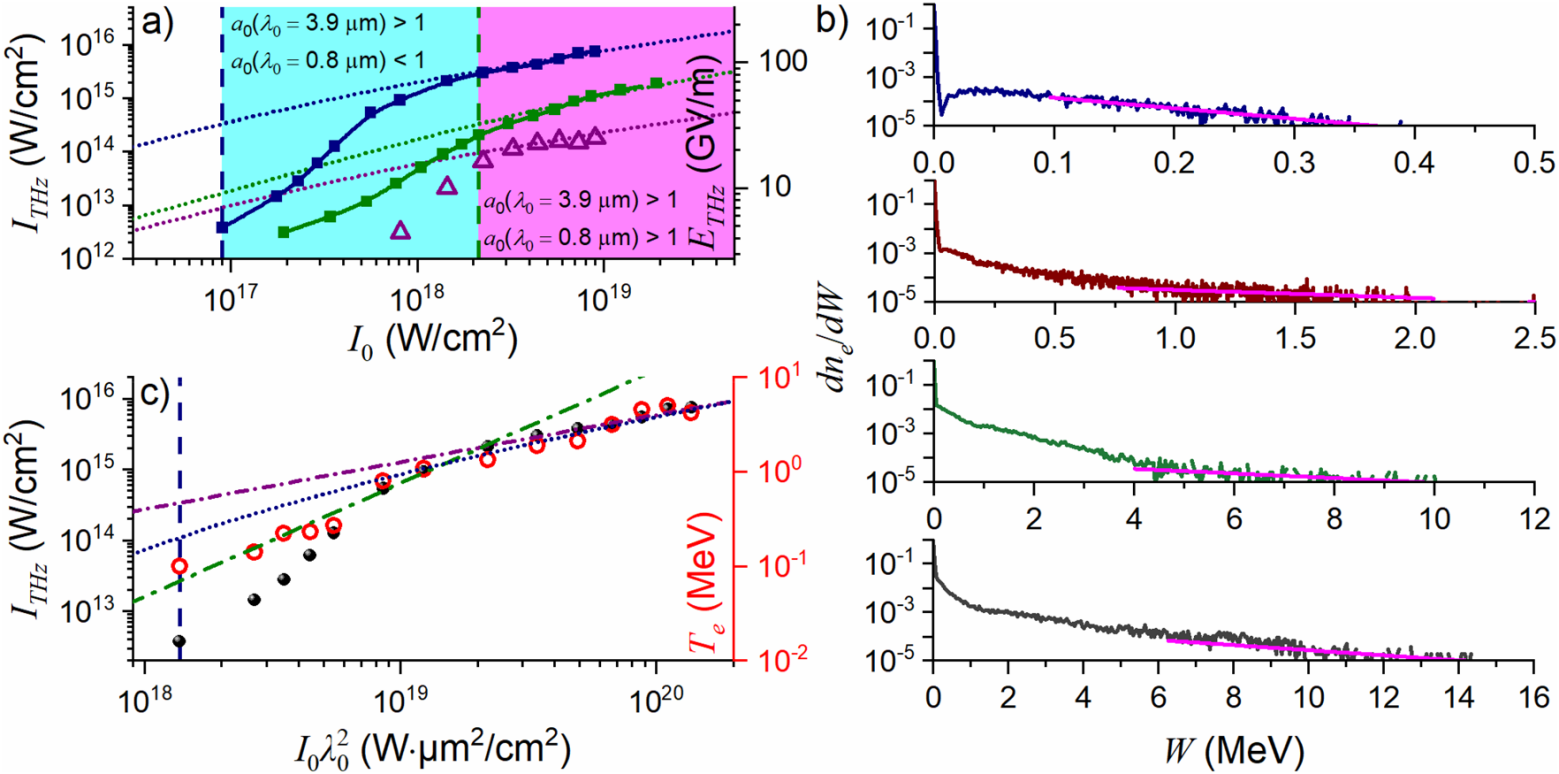
(**a**) The field strength (right axis) and field intensity (left axis) of THz radiation at *ξ* = 30*λ*_0_ as a function of the driver field intensity *I*_0_ for a *p*-polarized (green and blue boxes) and *s*-polarized (purple triangles) laser driver with *τ*_0_ = 80 fs and *λ*_0_ = 0.8 μm (green circles) and *λ*_0_ = 3.9 μm (blue boxes and purple triangles). Also shown is the ∝ Φ(*I*_0_*λ*_0_^2^) scaling, as dictated by the relativistic electron laser ponderomotive potential ${\mathscr{F}}_{{\text{e}}}$ for *λ*_0_ = 0.8 μm (green dotted lines) and *λ*_0_ = 3.9 μm (blue and purple dotted lines). (**b**) Energy distribution function of the recirculating electrons at the moment of time when these electrons reach the *y* = 0 plasma boundary versus the best Maxwellian fits of their high-energy tails (pink lines) for *I*_0_*λ*_0_^2^ increasing from top to bottom: *I*_0_*λ*_0_^2^ = 1.4 × 10^18^ W μm^2^/cm^2^ (blue curve), 2.2 × 10^19^ W μm^2^/cm^2^ (wine curve), 8.8 × 10^19^ W μm^2^/cm^2^ (green curve), and 1.4 × 10^20^ W μm^2^/cm^2^ (grey curve). (**c**) The field intensity of THz radiation at *ξ* = 30*λ*_0_ (left axis, blue filled circles) and the temperature of CTR-emitting electrons *T*_e_ (right axis, red open circles) as a function of *I*_0_*λ*_0_^2^. Also shown are the ∝ Φ(*I*_0_*λ*_0_^2^) scaling (blue dotted line) and the best (*I*_0_*λ*_0_^2^)^α^ fit for *T*_e_(*I*_0_*λ*_0_^2^) with *α* = 1.2 (green dash–dotted line) and 1/2 (purple dash–dotted line). Shown by dashed vertical lines are the driver intensities *I*_0_ (**a**) and the *I*_0_*λ*_0_^2^ product (**c**) corresponding to *a*_0_ = 1. Simulations are performed for a laser driver with two central wavelengths (*λ*_0_ = 0.8 and 3.9 μm), a pulse width *τ*_0_ = 80 fs, and a beam-waist diameter *d*_0_ = 4*λ*_0_. The laser slab has an initial electron density *n*_0_ = 4*n*_c_, a width *l*_*x*_ = 16*λ*_0_, and a depth *l*_*y*_ = 4*λ*_0_.

We now focus on the behavior of the THz yield in the considered laser–plasma setting as a function of the driver wavelength *λ*_0_ and field intensity *I*_0_. To this end, we set a detection point at *ξ* = 30*λ*_0_ along the *ξ*-axis in the direction of specular reflection (Fig. 1a, b, f) and define the field intensity as *I*_THz_ = [*c*/(2*μ*_0_)][*B*_0_(*ξ* = 30*λ*_0_)]^2^, where *B*_0_ is the peak *B*_*z*_ field at the trough of the first CTR wave (blue arrows in Fig. 3a, b) and *μ*_0_ is the magnetic permeability. In Fig. 5a, the THz intensity *I*_THz_ is plotted as a function of the driver field intensity *I*_0_ for two values of the driver wavelength, representing two types of relativistic-intensity short-pulse laser sources—Ti: sapphire lasers, *λ*_0_ = 0.8 μm^61,62^, and high-peak-power mid-infrared OPCPAs, *λ*_0_ = 3.9 μm^63–65^. Simulations here are performed for a laser driver with a pulse width *τ*_0_ = 80 fs and a beam-waist diameter *d*_0_ = 4*λ*_0_. The laser slab in these simulations has an initial electron density *n*_0_ = 4*n*_c_, a width *l*_*x*_ = 16*λ*_0_, and a depth *l*_*y*_ = 4*λ*_0_. The laser and plasma parameters *τ*_0_, *d*_0_, *n*_0_, *l*_*x*_, and *l*_*y*_ are thus defined in such a way that they change with the driver wavelength *λ*_0_. Specifically, the pulse width *τ*_0_ = 80 fs corresponds to ≈ 30 field cycles for a laser driver with *λ*_0_ = 0.8 μm and ≈ 6 field cycles for a *λ*_0_ = 3.9 μm driver.

For both driver wavelengths, the growth of the THz intensity as a function of *I*_0_ is seen to significantly slow down as the driver field intensity approaches the *a*_0_ = 1 relativistic borderline (the vertical dashed lines in Fig. 5a). Simulations performed within the range of laser pulse widths *τ*_0_ from 30 to 250 fs, initial electron densities *n*_0_ from 4 to 80*n*_c_, and plasma slab depths *l*_*y*_ from 0.8*λ*_0_ to 15*λ*_0_ show that, when varied within these ranges, neither of these parameters has any significant effect on the behavior of *I*_THz_ as a function of *I*_0_ and *λ*_0_ (Fig. 4e). In agreement with earlier studies^66,67^, an increase in *n*_0_ was found to give rise to a growth of computational noise.

To gain insights into this behavior of *I*_THz_(*I*_0_), we resort to the analysis of the energy distribution function of the recirculating electrons at the moment of time when these electrons reach the *y* = 0 plasma boundary. In Fig. 5b, we present such energy distribution functions for *I*_0_*λ*_0_^2^ ranging from 1.4 × 10^18^ W μm^2^/cm^2^ (*a*_0_ ≈ 1) to 1.4 × 10^20^ W μm^2^/cm^2^ (*a*_0_ ≈ 10). We then define the temperature of CTR-emitting electrons, *T*_e_, by fitting the high-energy tails of these functions with Maxwellian distribution functions (shown by the pink lines in Fig. 5b). Remarkably, in the limit of *a*_0_ > 1, the temperature *T*_e_ obtained via such a procedure tends to follow the same scaling as a function of *I*_0_*λ*_0_^2^ as the THz intensity *I*_THz_ (cf. blue filled circles and red open circles in Fig. 5c). Moreover, both *I*_THz_(*I*_0_*λ*_0_^2^) and *T*_e_(*I*_0_*λ*_0_^2^) are seen to converge in the high-*a*_0_ limit to the scaling of Φ(*I*_0_*λ*_0_^2^) = ${\mathscr{F}}_{{\text{e}}}$/(*m*_e_*c*^2^) (dotted line in Fig. 5a, c), as dictated by the laser pondermotive potential ${\mathscr{F}}_{{\text{e}}}$ = *m*_e_*c*^2^(*γ* − 1) of a relativistic electron with rest energy *m*_e_*c*^2^ and relativistic Lorentz factor *γ*.

To relate these tendencies in the behavior of the THz intensity to the physical picture of THz generation, we search for the best (*I*_0_*λ*_0_^2^)^α^ fit for *T*_e_(*I*_0_*λ*_0_^2^), allowing *α* to take different values below and above the *a*_0_ = 1 relativistic borderline. As shown in earlier studies^66–70^, for high, yet nonrelativistic driver intensities, with *a*_0_ ≤ 1 or *a*_0_ ~ 1, the temperature of hot electrons, *T*_e_, tends to scale as (*I*_0_*λ*_0_^2^)^α^, with *α* depending on the plasma gradient scale length and the incidence angle of the laser beam, varying from its lower bound at 1/3 to *α* > 1 in the case of laser vacuum heating^68,69^. However, for driver intensities well above the relativistic borderline of *a*_0_ = 1, the energy of laser radiation is absorbed predominantly via a ponderomotive acceleration of electrons, leading to a *T*_e_ ∝ (*I*_0_*λ*_0_^2^)^1/2^ scaling^69,70^. The *α* exponent is thus a meaningful probe that can help identify the physical scenario whereby THz radiation is generated as a part of ultrafast laser–plasma electrodynamics.

In Fig. 5c, we plot the intensity of THz radiation as a function of *I*_0_*λ*_0_^2^ along with two best (*I*_0_*λ*_0_^2^)^α^ fits for the *a*_0_ ~ 1 and *a*_0_ >  > 1 regimes. That the best (*I*_0_*λ*_0_^2^)^α^ fit for *I*_THz_(*I*_0_*λ*_0_^2^) near the *a*_0_ = 1 borderline (shown by dashed vertical lines in Fig. 5a, c) regime is achieved with *α* = 1.2 (green dash–dotted line) is indicative of the predominance of laser vacuum heating as a mechanism of laser electron acceleration in our laser–plasma setting. For *a*_0_ >  > 1, on the other hand, a search for the best (*I*_0_*λ*_0_^2^)^α^ fit for *I*_THz_(*I*_0_*λ*_0_^2^) converges to *α* = 1/2 (purple dash–dotted line), as expected for CTR-emitting electrons generated mainly by the laser ponderomotive force^66–70^. Both the electron temperature *T*_e_ and the THz yield are seen to monotonically increase with *λ*_0_ (Figs. 5a, 5c). At *I*_0_ ≈ 8 × 10^17^ W/cm^2^, the THz field in the trough of the first CTR wave induced by a *λ*_0_ = 3.9 μm driver is seen to be an order of magnitude higher than the respective THz field from laser plasmas driven by *λ*_0_ = 0.8 μm laser pulses (Fig. 5a). As the THz yield continues to grow with *I*_0_ in the *a*_0_ > 1 regime, THz fields with amplitudes above 0.2 MV/m can be generated from laser plasmas driven by mid-infrared pulses with a sufficiently high field intensity (*I*_0_ ≥ 7 × 10^18^ W/cm^2^ for *λ*_0_ = 3.9 μm in Fig. 5a). While the THz yield and the electron temperature *T*_e_ in subrelativistic, *a*_0_ < 1 plasmas with *L* ≤ *λ*_0_/20 follows the (*I*_0_*λ*_0_^2^)^α^ scaling with *α* > 1 (Fig. 5a, c), as an indication of laser vacuum heating^68,69^, for plasmas with significantly longer *L*, with *L* being as long as a few *λ*_0_, plasma electrons gain much of their energy via resonant absorption^40^, leading to *T*_e_ ∝ (*I*_0_*λ*_0_^2^)^α^ with *α* ≈ 1/3^40,66^. For strongly relativistic plasmas, on the other hand, with *a*_0_ >  > 1, both the THz yield and the electron temperature *T*_e_ are found to show little to no deviations from the (*I*_0_*λ*_0_^2^)^1/2^ scaling regardless of *L*.

While a *p*-polarized laser field can drive plasma electrons via both the ponderomotive force and vacuum heating, an *s*-polarized driver does not provide an effective field component that would induce vacuum-heating acceleration. The intensity of THz radiation from laser plasmas driven by a moderate-*a*_0_ *s*-polarized laser pulse is therefore much lower (purple triangles in Fig. 5a) than the intensity of THz radiation generated by a *p*-polarized driver with the same *a*_0_ (blue boxes in Fig. 5a). Yet, in the high-*a*_0_ regime, where a ponderomotive acceleration dominates over vacuum heating, *I*_THz_(*I*_0_*λ*_0_^2^) is seen to converge to the scaling of Φ(*I*_0_*λ*_0_^2^) = ${\mathscr{F}}_{{\text{e}}}$/(*m*_e_*c*^2^) (blue and purple dotted lines in Fig. 5a) regardless of the polarization of the driver.

## Three-dimensional analysis

**Figure 6:**
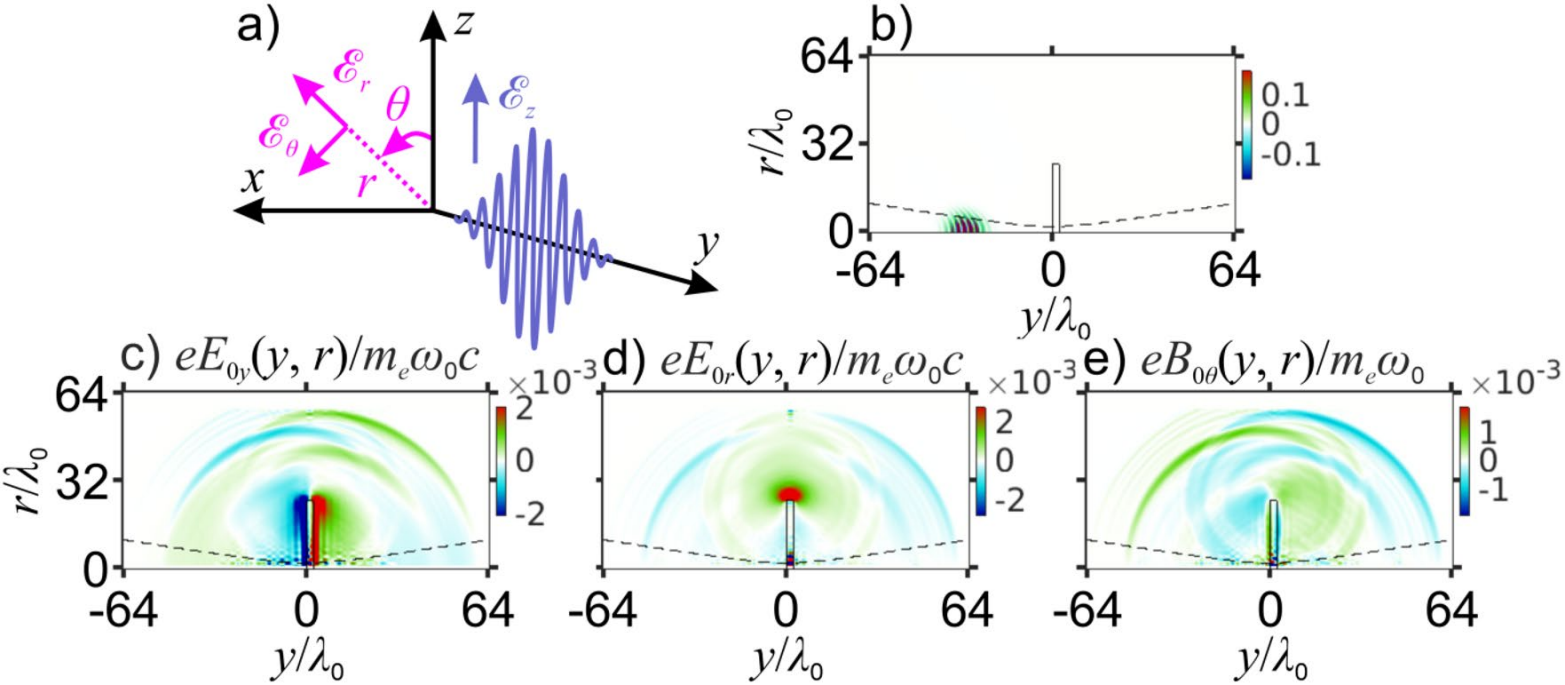
(**a**) The driving laser field (blue line) and its components against the Cartesian (black) and cylindrical coordinates (pink) used in 3D PIC simulations. (**b**) An *ry*-map of the laser field approaching the plasma target. (**c**–**e**) Spatial distributions of the *E*_0*y*_(*y*, *r*) (**c**), *E*_0*r*_(*y*, *r*) (**d**), and *B*_0*θ*_(*y*, *r*) (**e**) components of the THz field for *t* = 90*T*_0_ and *a*_0_ = 1. The dashed line is the FWHM radius of the driver laser beam propagating in space with no plasmas. Also shown is laser-driven electric charge separation in shielding plasma currents (red and blue coding is for the positive and negative charges, respectively), giving rise to antenna-like emission of THz radiation from plasma edges.

The purpose of the analysis presented in this section is to show that the key tendencies in the behavior of the THz laser-plasma output as a function of the parameters of the laser driver, such as *I*_0_ and *λ*_0_, are in no way limited to the specific 2D oblique-incidence laser–plasma interaction geometry examined in the previous sections, but should be viewed instead as signatures of laser-driven plasma electrodynamics representative of a vast, rather general class of laser–plasma interaction settings. To reach this goal, we resort to a 3D PIC modeling of laser–plasma interactions driven by a relativistic-intensity laser driver propagating along the normal to the plasma surface. Due to its cylindrical symmetry, the field of such a driver can be decomposed into azimuthal modes^53^. With the *y*-axis chosen along the field propagation direction (Fig. 6a, b), as in the previous sections, a laser-driver field polarized along the *z*-axis is recognized as a pure azimuthal mode of order *m* = 1. Such a field is conveniently described in cylindrical coordinates *r*, *θ*, *y* (Fig. 6a) and is fully defined by *m* = 1 azimuthal-mode components ${\mathscr{E}}$_1*y*_(*y*, *r*), ${\mathscr{E}}$_1*r*_(*y*, *r*), ${\mathscr{E}}$_1*θ*_(*y*, *r*), ${\mathscr{B}}$_1*y*_(*y*, *r*), ${\mathscr{B}}$_1*r*_(*y*, *r*), and ${\mathscr{B}}$_1*θ*_(*y*, *r*). As such a laser field drives ultrafast electron oscillations within a plasma target, it gives rise to a pure *m* = 0 azimuthal-mode of THz radiation^53^ with cylindrical field components *E*_0*y*_(*y*, *r*), *E*_0*r*_(*y*, *r*), *E*_0*θ*_(*y*, *r*), *B*_0*y*_(*y*, *r*), *B*_0*r*_(*y*, *r*), and *B*_0*θ*_(*y*, *r*). The azimuthal-mode decomposition of the fields and electron currents for laser–plasma interactions of such a symmetry thus helps to substantially reduce the computation complexity of the problem while preserving its 3D nature^53,71^. The plasma target in these simulations is taken in the form of a *l*_*y*_ x *l*_*r*_ = 2.5*λ*_0_ × 25*λ*_0_ cylinder with *n*_*e*_ = 5*n*_*c*_ (Fig. 6b), supporting the overall cylindrical symmetry of the problem.

**Figure 7:**
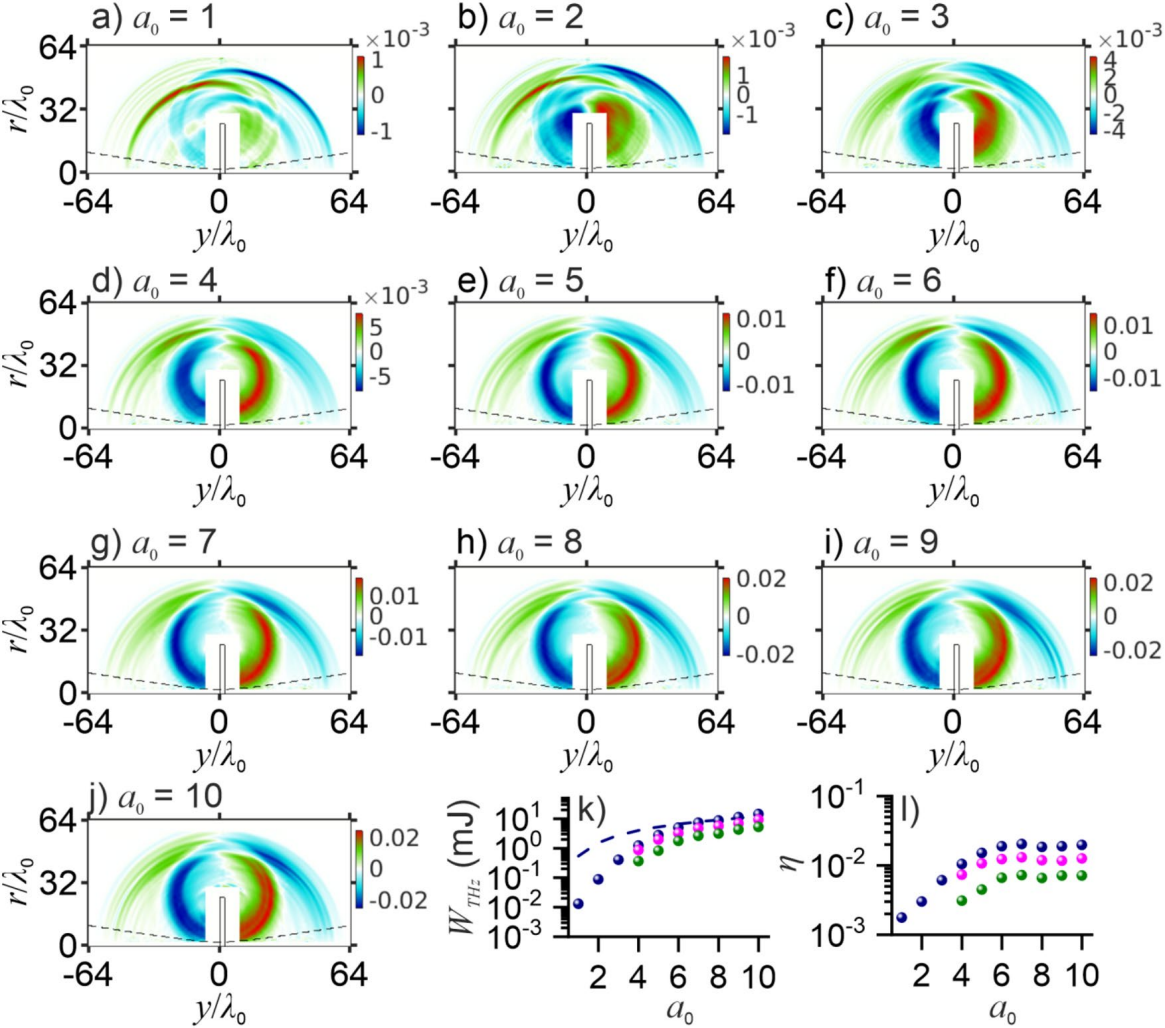
(**a**–**j**) Spatial distributions of the THz field *B*_0*θ*_(*y*, *r*) at *t* = 85*T*_0_ for *a*_0_ as specified in the panels. The dashed line is the FWHM radius of the driver laser beam propagating in space with no plasmas. (**k**, **l**) THz output energy (**k**) and laser-to-THz energy conversion efficiency (**l**) as functions of *a*_0_: (green circles) the CTR component, (pink circles) antenna-like plasma emission, and (blue circles) overall THz output. The ∝ Φ(*I*_0_*λ*_0_^2^) scaling is shown by the dashed line.

In Fig. 6c–e, we present typical *ry*-maps of the *E*_0*y*_(*y*, *r*), *E*_0*r*_(*y*, *r*), and *B*_0*θ*_(*y*, *r*) field components in the THz laser—plasma output. Clearly seen in these maps are two forward-propagating THz CTR waves, emitted by the rear plasma boundary, and one backward THz CTR wave, radiation by the front plasma boundary. Also readily discernible are spherical THz waves emitted by plasma edges. Radiation of THz waves of this class has been earlier identified as antenna-like emission by shielding electron currents flowing along the plasma surfaces as a part of laser-driven ultrafast charge-carrier dynamics inside the plasma^31^. Charge separation arising as a result of such dynamics is shown by red and blue coding in Fig. 6c–e. Similar to electric currents in an impulsively driven antenna^72^, such electron currents provide a source of outgoing spherical waves of electromagnetic radiation (clearly seen in Fig. 6c–e, as well as in Fig. 7a–j) as they are brought to a halt near the edges of the plasma target^31^.

Shown in Fig. 7a–j are the *ry*-maps of the *B*_0*θ*_(*y*, *r*) field component in THz radiation from the plasmas driven by a laser field with the laser vector potential ranging from *a*_0_ = 1 in Fig. 7a to = 10 in Fig. 7j. As *a*_0_ increases, antenna-like THz emission of laser-driven plasmas is seen to become more and more isolated in space from THz CTR waves, allowing an accurate separation of radiation energies, *W*_1_ and *W*_2_, in the antenna-emission and CTR-wave components of the THz laser-plasma output. In Fig. 7k, we present these energies along with the total energy of the THz output, *W*_THz_ = *W*_1_ + *W*_2_, as functions of *a*_0_. The THz output energy is seen to grow with *a*_0_, reaching the level above 10 mJ for *a*_0_ ~ 10.

The key tendencies in the behavior of *W*_THz_ as a function of *a*_0_, are fully consistent, as a comparison of Figs. 5a, b, 7k, l shows, with the behavior of the THz radiation output in the 2D laser–plasma interaction geometry. Similar to the 2D laser–plasma setting, THz radiation in 3D simulations is emitted in both forward direction and in reflection. However, because the THz energy cannot be calculated in a 2D setting, where one dimension is missing, 2D scaling laws are formulated for the THz intensity rather than the THz energy. In the 3D setting, on the other hand, where the full THz radiation energy *W*_THz_ can be calculated, analysis of the THz energy as a function of *I*_0_ and *λ*_0_ becomes possible.

Similar to the THz output in 2D simulations (Fig. 5a, b), the THz energy *W*_THz_ in Fig. 7k is seen to rapidly grow with *a*_0_ in the range of moderate *a*_0_, indicating vacuum heating as a predominant physical mechanism whereby the energy of the laser driver is transferred to THz radiation via electron acceleration. As *a*_0_ grows above the *a*_0_ > 1 level, *W*_THz_ slows its growth, converging to a much more gently sloping Φ(*I*_0_*λ*_0_^2^) asymptotics (shown by the dashed line in Fig. 7k), thus showing once again a close similarity with 2D simulations (cf. Figures 5a, b and 7k). As the laser-driver field approaches the *a*_0_ ~ 10 level, *W*_THz_(*a*_0_) follows the ∝ ${I}_{0}^{1/2}{\lambda }_{0}$ scaling with a very high accuracy, indicating, in full agreement with the 2D analysis, the decisive role of relativistic ponderomotive charge acceleration as a physical mechanism behind laser-to-THz energy conversion.

## Conclusion

To summarize, we have shown that, while the *I*_0_ ${\lambda }_{0}^{2}$ product of the laser intensity *I*_0_ and the laser wavelength *λ*_0_ plays the key role in the energy scaling of strong-field laser-plasma THz generation, the THz output energy, *W*_THz_, does not follow the *I*_0_ ${\lambda }_{0}^{2}$ scaling. Its behavior as a function of *I*_0_ and *λ*_0_ is instead much more complex. Our two- and three-dimensional PIC analysis shows that, for moderate, subrelativistic and weakly relativistic fields, *W*_THz_(*I*_0_ ${\lambda }_{0}^{2}$) can be approximated as (*I*_0_*λ*_0_^2^)^*α*^, with a suitable exponent *α*, as a clear signature of vacuum electron acceleration as a predominant physical mechanism whereby the energy of the laser driver is transferred to THz radiation. For strongly relativistic laser fields, on the other hand, *W*_THz_(*I*_0_ ${\lambda }_{0}^{2}$) closely follows the scaling dictated by the relativistic electron laser ponderomotive potential ${\mathscr{F}}_{{\text{e}}}$, converging to *W*_THz_ ∝ ${I}_{0}^{1/2}{\lambda }_{0}$ for very high *I*_0_, thus indicating the decisive role of relativistic ponderomotive charge acceleration as a mechanism behind laser-to-THz energy conversion. Analysis of the electron distribution function shows that the temperature of hot laser-driven electrons bouncing back and forth between the plasma boundaries displays the same behavior as a function of *I*_0_ and *λ*_0_, altering its scaling from (*I*_0_*λ*_0_^2^)^*α*^ to that of ${\mathscr{F}}_{{\text{e}}}$, converging to *W*_THz_ ∝ ${I}_{0}^{1/2}{\lambda }_{0}$ for very high *I*_0_. Specifically, as can be seen from the comparison of THz yields attainable from laser-plasma sources driven with 0.8- and 3.9-μm laser pulses (green and blue boxes in Fig. 5a along with the respective Φ(*I*_0_*λ*_0_^2^) asymptotes), the use of a 3.9-μm, sub-100-fs output of the latest-generation high-power OPCPAs^37–39,63–65^ can significantly enhance THz generation from relativistic laser–plasma settings relative to plasmas driven by standard, 0.8-μm Ti: sapphire laser pulses. Relativistic field intensities have been already achieved for such sources^63,64^. The work to even higher *I*_0_ is in progress. These findings provide a clear physical picture of THz generation in relativistic laser plasmas, suggesting the THz yield *W*_THz_ resolved as a function of *I*_0_ and *λ*_0_ as a meaningful measurable that can serve as a probe for the temperature *T*_e_ of hot electrons in a vast class of laser–plasma interactions.

## Data Availability

All data generated or analyzed during this study are included in this published article.
